# Pre-course online cases for the world health organization's basic emergency care course in Uganda: A mixed methods analysis

**DOI:** 10.1016/j.afjem.2022.03.005

**Published:** 2022-04-20

**Authors:** Alexandra Friedman, Lee A. Wallis, Julia C. Bullick, Charmaine Cunningham, Joseph Kalanzi, Peter Kavuma, Martha Osiro, Steven Straube, Andrea G. Tenner

**Affiliations:** a School of Medicine, University of California San Francisco, Zuckerberg San Francisco General Hospital, San Francisco, California, United States of America; b Division of Emergency Medicine, University of Cape Town, Bellville, South Africa; c Department of Emergency Medicine, Northwest Permanente, Clackamas, Oregon, United States of America; d Division of Emergency Medicine, Makerere University, Mulago Hill Road, Kampala, Uganda

**Keywords:** Emergency, Blended learning, Low resource, Short courses

## Abstract

**Introduction:**

The Ministry of Health - Uganda implemented the World Health Organization's Basic Emergency Care course (BEC^1^) to improve formal emergency care training and address its high burden of acute illness and injury. The BEC is an open-access, in-person, short course that provides comprehensive basic emergency training in low-resource settings. A free, open-access series of pre-course online cases available as downloadable offline files were developed to improve knowledge acquisition and retention. We evaluated BEC participants’ knowledge and self-efficacy in emergency care provision with and without these cases and their perceptions of the cases.

**Methods:**

Multiple Choice Questions (MCQs^2^) and Likert-scale surveys assessed 137 providers’ knowledge and self-efficacy in emergency care provision, respectively, and focus group discussions explored 74 providers’ perceptions of the BEC course with cases in Kampala in this prospective, controlled study. Data was collected pre-BEC, post-BEC and six-months post-BEC. We used liability analysis and Cronbach alpha coefficients to establish intercorrelation between categorised Likert-scale items. We used mixed model analysis of variance to interpret Likert-scale and MCQ data and thematic content analysis to explore focus group discussions.

**Results:**

Participants gained and maintained significant increases in MCQ averages (15%) and Likert-scale scores over time (p < 0.001). The intervention group scored significantly higher on the pre-test MCQ than controls (p = 0.004) and insignificantly higher at all other times (p > 0.05). Nurses experienced more significant initial gains and long-term decays in MCQ and self-efficacy than doctors (p = 0.009, p < 0.05). Providers found the cases most useful pre-BEC to preview course content but did not revisit them post-course. Technological difficulties and internet costs limited case usage.

**Conclusion:**

Basic emergency care courses for low-resource settings can increase frontline providers’ long-term knowledge and self-efficacy in emergency care. Nurses experienced greater initial gains and long-term losses in knowledge than doctors. Online adjuncts may enhance health professional education in low-to-middle income countries.

## African relevance

• Short courses may address a significant gap in health worker training in basic emergency care in Sub-Saharan Africa.
• Online open educational resources could enhance healthcare worker education in Sub-Saharan Africa as Internet access expands and costs decrease.
• Nurses and doctors show differential knowledge retention in blended short courses that may require targeted educational strategies.

## Introduction

Formal emergency care could directly address over half of all deaths and a third of all disability in low-to-middle-income countries (LMICs)[1]. Most LMIC emergency care systems lack dedicated investment due to the prioritisation of vertical programs addressing specific diseases[2]. This contributes to a lack of formal emergency care training in regions like Sub-Saharan Africa (SSA), where 3% of the world's healthcare workers (HCWs) confronts 24% of the world's disease burden[3]. Short courses have emerged as a high-impact intervention to bolster emergency-specific skills and knowledge among HCWs in LMICs where formal, long-term training options will not fill the training gap in the next decade[4], [5], [6].

Blended learning models combine in-person and online learning, which may enhance short courses for HCWs in LMICs[7], [8], [9]. Blended short courses incorporate open-access digital educational materials, i.e. open educational resources (OERs), to improve information access and knowledge while reducing course costs, materials and faculty burden[7,10]. Blended learning requires substantial implementation efforts to reach efficacy in low-resource settings[7,11,12].

Responding to these challenges, the World Health Organization (WHO) created the Basic Emergency Care Course (BEC), the first short course with open-access materials and no participant fees to provide comprehensive basic emergency training for low-resource settings. The five-day course caters to all frontline HCW cadres and covers high-yield modules using lectures, discussions and skills practicums (available at https://www.who.int/publications/i/item/basic-emergency-care-approach-to-the-acutely-ill-and-injured). Following the BEC's successful multi-country pilot[13], the University of California San Francisco (UCSF) WHO Collaborating Centre for Emergency and Trauma Care developed OERs for the BEC, including 32 pre-course clinical cases covering all BEC learning points (available at https://emergencycare.ucsf.edu/basic-emergency-care-course-adjuncts), to improve knowledge acquisition and retention. The Centre conducted OER feasibility and acceptability pilots at two small Tanzanian sites, but a larger study was needed to evaluate their efficacy and utility.

Uganda is a low-income East African nation with a high burden of infectious disease and trauma, including one of the world's highest rates of road traffic collisions[14,15]. Its nascent emergency care system exists within a tiered, decentralised healthcare model with few 24-hour dedicated emergency centres and limited ambulance services[16,17]. Ugandan hospitals rely on outpatient clinics and inpatient wards to provide emergency care: 70% of hospitals lack the basic infrastructure to do so[18,19]. The Ministry of Health (MoH) Department of Emergency Medical Services implemented the BEC course at select hospitals with high emergency volumes across Uganda, including in the capital Kampala, to train emergency providers.

We conducted a mixed-methods, prospective cohort study to investigate the BEC's impact with pre-course cases on BEC participants’ knowledge and self-efficacy in emergency care provision and to explore participants’ perceptions of the course and cases. We hypothesised that pre-course case exposure would enhance participants’ pre-course knowledge acquisition and post-course knowledge retention.

## Methods

Three regional referral, one national referral and two private-not-for-profit hospitals with high acuity in Kampala were selected as study sites by the MoH. Study participants included hospital-based nurses, midwives, clinical officers and doctors from various departments who regularly managed emergency patients.

We recruited 142 providers for the BEC courses and study by convenience sampling based on hospital administrators’ and colleagues’ recommendations, and instructed providers from four of the six hospitals to complete at least half of the pre-course cases before the course. Thirty-two cases covering all BEC teaching points were available online or as downloadable offline files. Participants were assigned to the intervention group, i.e. OER group that completed online cases before the BEC, or the control group, i.e. the group that did not receive online cases before the BEC. Both groups completed the BEC. Group assignment was based on the course's timing with later courses assigned to the control group.

The study staff undertook extensive implementation steps to facilitate case usage, including teaching on-site sessions, calling individuals, and distributing flash drives containing the cases in offline form to each site. We recruited a 46-participant subset for post-BEC focus group discussions (FGDs) by random sampling and re-invited them for the six-months post-BEC FGDs, along with 28 additional participants recruited by convenience sampling to account for attrition. Each FGD consisted of four to nine participants per course for a total of 74 study participants.

Participants completed a standardised 25-item MCQ designed by the course creators to assess basic emergency care knowledge pre-BEC in-person course, post-BEC in-person course and six-months post-BEC. The OER group received access to the cases and instructions to complete them before the pre-test. We designed and administered a ten-item Likert survey on a four-point scale to assess BEC participants’ self-efficacy in emergency care provision with items grouped to measure comfort, knowledge, confidence and preparedness.

We used mixed model analysis of variance (ANOVA) to assess self-efficacy and knowledge in emergency care, excluding clinical officers given small sample size (n=9). We grouped providers by OER exposure and cadre, and treated modality and time effects as fixed and participant effects as random. A post-hoc subgroup analysis assessed the effect of self-reported case completion. We used liability analysis to establish intercorrelation between the Likert categories using Cronbach alpha coefficients. A coefficient >0.60 signified intercorrelation given the limited item numbers per category. We then applied mixed model ANOVA to assess each category's relationship with time, pre-course case exposure and cadre.

We designed semi-structured FGD scripts based on similar studies to explore providers’ perceptions of the course and cases post-BEC and six-months post-BEC[20], [21], [22], [23]. The FGD number needed for thematic saturation was estimated based on senior investigators’ experience. The course coordinator AF, a U.S. medical student with a qualitative background, facilitated and recorded FGDs in the national language of English in private rooms at each site. Participants knew AF before the FGDs and she emphasised her removed role in the BEC before every FGD. The FGDs averaged 45 minutes and only participants and researchers were present. One Ugandan researcher attended the post-BEC FGDs to take notes, transcribed all post-BEC audio-recordings and translated local phrases. AF transcribed all six-months post-BEC FGDs audio-recordings. All identifiers were removed and the recordings were deleted.

AF and CC conducted a thematic content analysis of the FGD transcripts, independently coding content into themes and sub-themes. They compared, discussed and refined their analyses in an iterative process, triangulating findings and discrepancies with senior investigators and the quantitative data, until reaching agreement and thematic saturation for both rounds. AF used Atlas.ti 8™ and CC used manual analysis to code data into themes and sub-themes.

The study team collected written informed consent from all participants before the course and before the FGDs. The University of Cape Town and Makerere University's Human Research Ethics Committees and UCSF's Institutional Review Board provided ethical approvals, respectively 330/2018, 2018-117 and 18-24418, for the study.

## Results

We enrolled 142 participants and included 137 in the quantitative analysis, excluding five participants who did not complete the course. Most participants were nurses (including registered, diploma, midwives and nurse assistants) followed by doctors (including interns, general physicians and specialists) and advanced practice, non-physician providers known as clinical officers who manage patients with a supervising physician (Table 1). Eighty-six participants in the OER group received instructions to complete at least 16/32 pre-course cases online or as downloadable offline files, and 51 participants in the control group did not.

**Table 1:** BEC participants in OER and control groups by cadre

	OER Group	Control Group	TOTAL (n,%)
Nurses	51	36	87, (63%)
Clinical Officers	8	2	9, (7%)
Doctors	27	13	41, (30%)
TOTAL (n, (%))	86, (63%)	51, (37%)	137, (100%)

BEC, Basic Emergency Care course; OER, open educational resources

Most providers (99%) completed pre- and post-course MCQs and Likert scales, and 110 (80%) completed six-month follow up MCQs and Likert scales. Sixty-five OER participants (76%) self-reported completing at least one case and 35 participants (41%) self-reported completing ≥ 16 cases. OER participants averaged 10 completed cases.

**Fig. 1:**
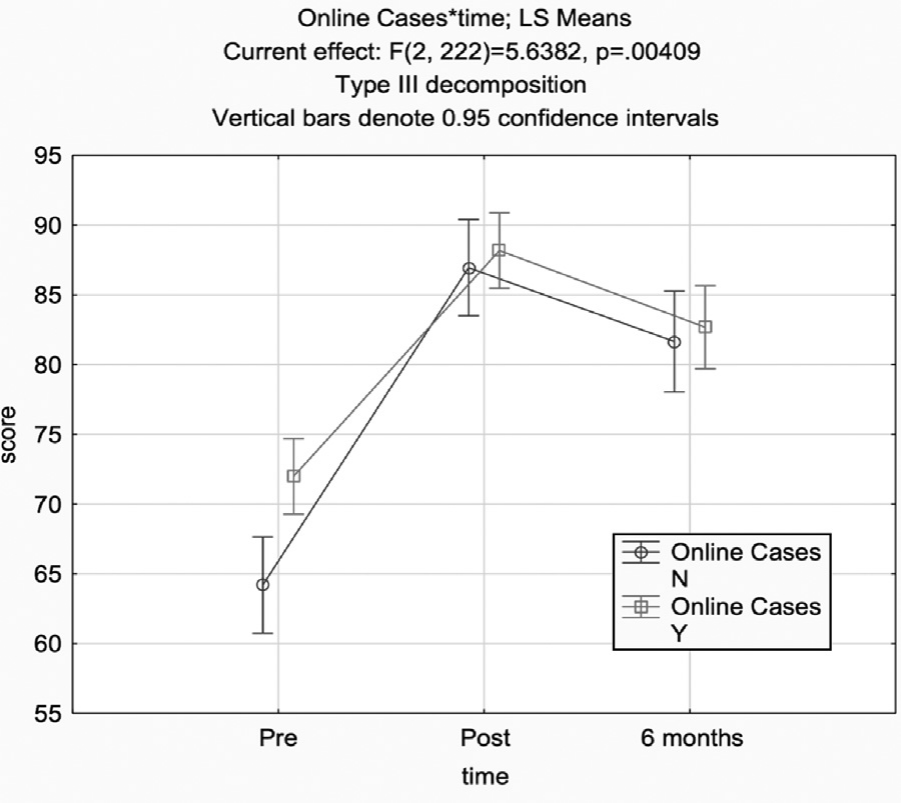
Online case assignment and time in ANOVA of MCQ scores

Participants’ mean MCQ scores increased from 66% pre-test to 86% post-test and decreased to 80% six-months post-BEC (Table 2). The average score increased by five correct answers (20%) from pre-test to post-test, and four correct answers (15%), from pre-test to six months post-BEC (Table 3). Participants experienced an average reduction of one incorrect answer (5%) from post-test to six-months post-BEC tests. ANOVA revealed a significant relationship between OER group assignment and time (p=0.004). The OER group had greater mean MCQ scores than the control group at all time points, though this relationship was only statistically significant on the pre-test (70.0% versus 60.0%, p = 0.004, Fig. 1).

**Table 2:** Average MCQ score by composite, OER and control groups and based on self-reported case completion in OER group

	Pre-BEC	Post-BEC	6 months post-BEC
MCQ composite average score (%)*	60.5, 68.8 **(65.7)**	84.9, 86.0 **(85.6)**	79.2, 81.0 **(80.3)**
Composite standard deviation for MCQ average score	14.7	11.0	12.8
MCQ average score for self-reported assignment completion (16 cases, n = 35)	72.5	88.4	85.6
MCQ average score for no self-reported case completion (n = 21)	62.2	79.6	76.7

MCQ, Multiple Choice Questions; OER, open educational resources, BEC, Basic Emergency Care course; *, Values reported as: control group, open educational resources group **(composite)**

**Table 3:** Average change in MCQ and Likert scores over time

	Δ Pre to Post	Δ Pre to 6 months	Δ post to 6 months
Average MCQ Δ in total score (%)*	24.4, 17.2 **(19.9)**	18.7, 12.2 **(14.6)**	-5.7, -5.0 **(-5.3)**
Average Likert Δ in total score*	7, 6.8 **(6.5)**	5.1, 4.3 **(4.8)**	-1.2, -1.4 **(-1.2)**
Median Likert Δ in total score*	6.0, 7.0 **(7.0)**	4.0, 4.0 **(4.0)**	-1.0, -1.0 **(-1.0)**

MCQ, Multiple Choice Questions; *, Values reported as: control group, open educational resources group **(composite)**

A post-hoc sub-group analysis assessing self-reported case completion showed no significant dose-response relationship between number of cases completed and MCQ score, and no significance between assignment completion and MCQ score. The “assignment completion” group (i.e. pre-course completion of  ≥ 16 cases) had insignificantly higher average MCQ scores at all time points than the controls and the “no case completion” group (Table 2). The controls scored higher on the post-test and six-month post-BEC test than the no case completion group. The mean score difference was greatest between the assignment completion and no case completion groups.

**Fig. 2:**
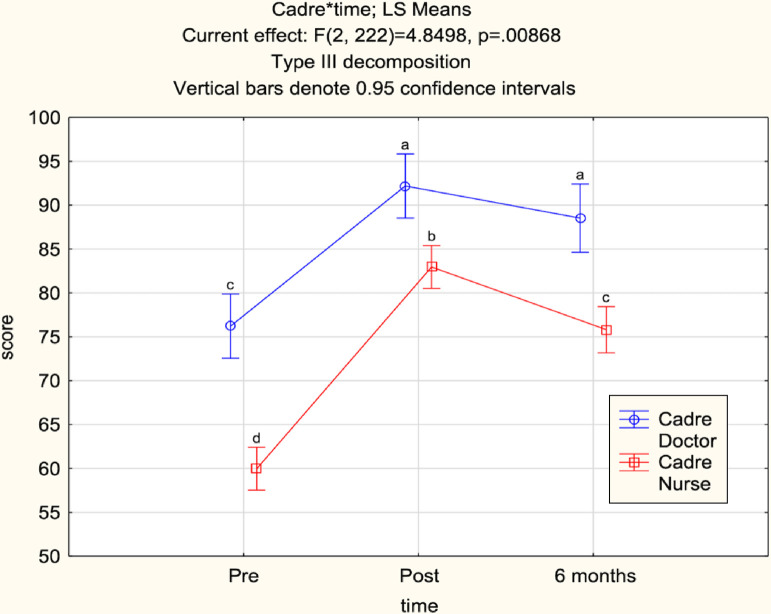
Cadre and time in ANOVA analysis of MCQ scores.

ANOVA showed a significant relationship between cadre and time on MCQ score (p = 0.009) with significant differences in nurses’ and doctors MCQ scores at all time points (p<0.001) (Fig 2). In comparison to doctors, nurses averaged a lower pre-BEC baseline score (61% versus 77%), gained significantly more knowledge from the pre-test to post-test, and retained significantly less knowledge from post-test to six-months post-BEC (p<0.001, p=0.07) (Fig 2, Table 4). Both doctors and nurses demonstrated significant knowledge retention (p<0.001) from the pre-test to six-months post-BEC test with a mean score improvement of 12% for doctors and 16% for nurses.

Letters denote significant differences i.e. no overlapping of confidence intervals between comparison points.

**Table 4:** Mean MCQ score differences by cadre and time excluding clinical officers (n=127)

1^st^ Mean	2^nd^ Mean	Mean Difference	Standard Error	p-value
Doctor pre-test	Doctor post-test	16.0	1.9	0
Doctor pre-test	Doctor 6 months post-test	12.3	2.0	0
Doctor post-test	Doctor 6 months post-test	-3.7	2.0	.07
Nurse pre-test	Nurse post-test	23.0	1.3	0
Nurse pre-test	Nurse 6 months post-test	15.8	1.3	0
Nurse post-test	Nurse 6 months post-test	-7.1	1.3	0
Doctor pre-test	Nurse pre-test	-16.2	2.2	0
Doctor post-test	Nurse post-test	-9.2	2.2	0
Doctor 6 months post-test	Nurse 6 months post-test	-12.7	2.4	0

Likert scale scores measuring self-efficacy in emergency care provision significantly increased in the composite group post-BEC with retention at six months and no significant differences between the OER and control groups (Table 3). All participants experienced a mean 16% increase in score from pre to post-BEC and 12% increase in score from pre-BEC to six months. The OER group had insignificantly higher average scores at all time points than the control group.

A liability analysis measured Cronbach's alpha coefficients to test intercorrelation between item groupings (Table 5) later applied to mixed model ANOVA. No coefficient was calculated for one item measuring confidence in colleagues. The items measuring “knowledge”, “confidence” and “preparedness” were inter-correlated. Two items measuring comfort in patient care were correlated, but comfort in following protocol was not. “Comfort” therefore best approximated comfort in patient care and had low overall intercorrelation. Two “confidence” items were considered intercorrelated based on equivalent item-total correlation despite a Cronbach alpha coefficient <0.60.

**Table 5:** Likert liability analysis results by category

Category	Cronbach alpha coefficient	Average inter-item correlation
Comfort	0.42	0.2
Confidence in colleagues	Not applicable	Not applicable
Knowledge	0.62	0.4
Preparedness	0.74	0.5
Confidence	0.48	0.3

**Fig. 3:**
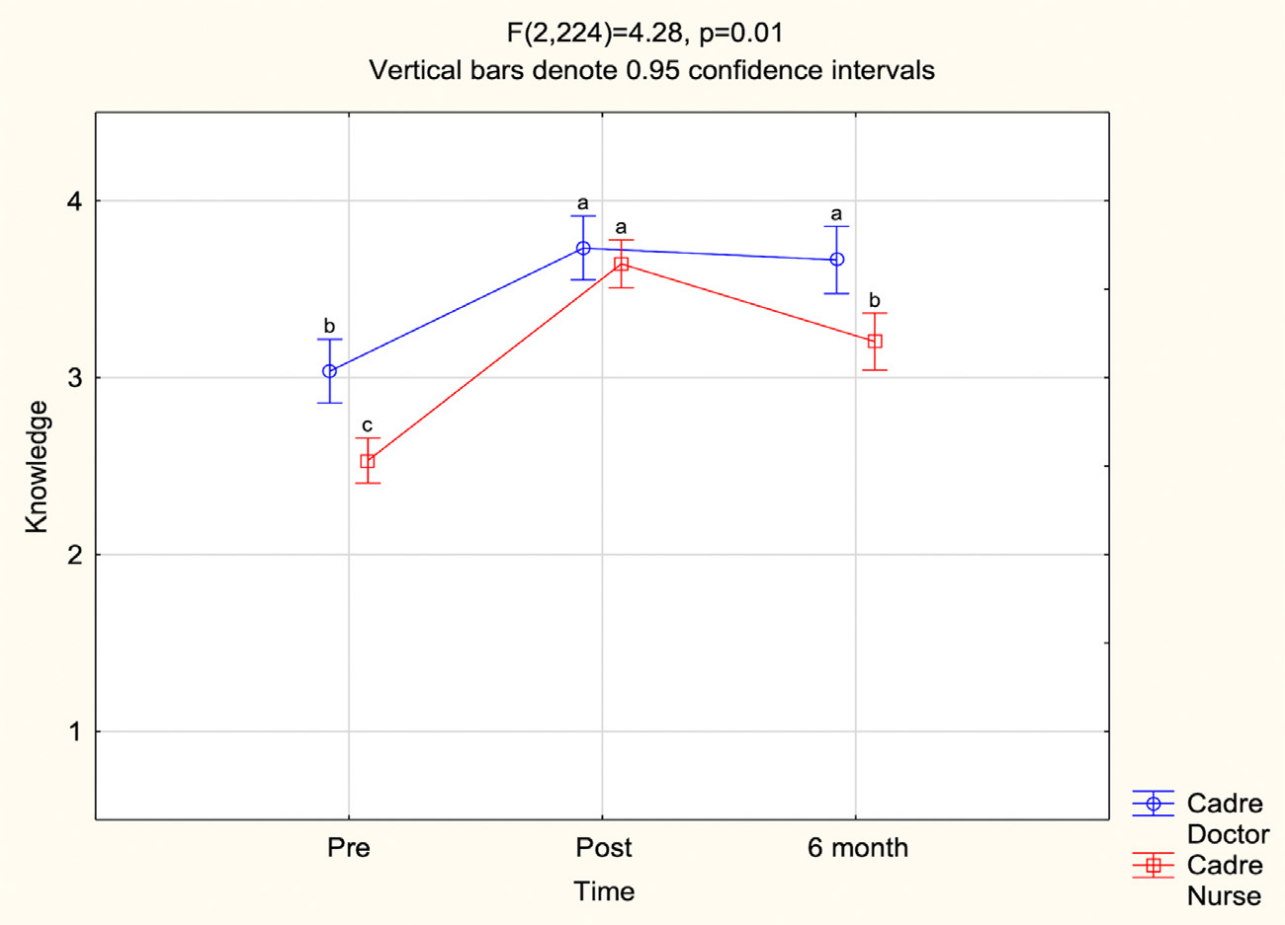
Y-axis is “Knowledge”, X axis is “Time”.

**Fig. 4:**
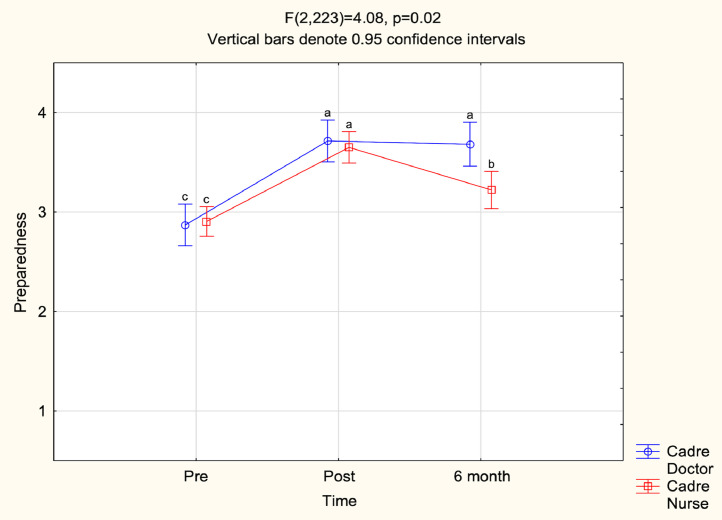
Y-axis is “Preparedness”, X axis is “Time”.

ANOVA assessed the validated groupings of self-rated knowledge, confidence, comfort in emergency care provision and preparedness. Together cadre and time significantly impacted self-ratings of “knowledge” and “preparedness” (Figs. 3 and 4). Compared to doctors, nurses’ self-ratings of knowledge and preparedness were lower pre-BEC, equivalent post-BEC, and significantly lower six-months post-BEC (p = 0.01) whereas doctors maintained elevated self-ratings with insignificant reductions. “Confidence” correlated with time and cadre individually, but not together. “Confidence in colleagues” did not significantly change. Assignment completion did not impact participants’ pre-BEC Likert-item scores in the post-hoc subgroup analysis.

We enrolled a 74-participant subset in two rounds of FGDs conducted post-BEC and six-months post-BEC at each site. Most participants (n=30, 61%) self-reported having no formal emergency care training though had worked in dedicated emergency, intensive care, and obstetric units. Most doctors (n = 4/7) and one-quarter of nurses and clinical officers (n = 9/39) had completed modular trainings on emergency topics. None reported formal emergency care training within their undergraduate professional education.

Most OER group participants (n=21, 70%) self-reported completing at least one case and 43% (n=13) self-reported completing ≥ 16 cases in the post-BEC FGDs. Participants who completed cases stated that they piqued interest and set expectations for the course. They found the cases applicable, satisfactory in quality, and effective in their real-time explanations of wrong and right answers. Few participants anticipated using the cases as teaching, reference or study tools post-BEC. Most self-reported minimal case usage in the follow-up FGDs.

The required investments of time and money for case completion limited pre-course and post-BEC case usage despite the cases’ free, open-access nature. Explicit barriers included time constraints, technological difficulties, poor or absent network connectivity, and lack of smart phone or computer. Nurses reported more difficulties and less access to technology than doctors. The most significant implicit barrier to case usage was motivation to undertake uncompensated and uncredited independent learning. One provider summarised,

*“Because there are people who left school long ago and they are not abreast with the current things, so there is that challenge…time is short… with adult learning, what motivates them? It is until you turn to class [for motivation] but right now as we still have them [the cases], we shall use them as training materials.”* [Clinical Officer, FGD2]

Every follow-up FGD suggested and favoured creating CME-accredited, internal BEC trainings to enhance knowledge retention and incentivise continued learning over independent OER usage.

The most salient BEC modules were the ABCDE approach, difficulty in breathing and airway management. Providers valued the skills practicums and general frameworks over discrete topics. A minority felt the ABCDE framework had limited value in resource-constrained settings. One provider explained, “*you manage a patient… [knowing] you would have taken the patient through the ABCDE procedures, but your hands are tied and always to me, my patients end up dying.”* [Clinical Officer, FGD2]

Participants believed that the course improved patient outcomes through a “*common understanding*” [Doctor, FGD12] that enabled teamwork among providers. One emergency nurse estimated that due to his team's BEC training, *“At least 70% of them [patients] who would have died are making it.”* [Nurse, FGD9] A surgeon described introducing a BEC-based *“morbidity and mortality audit”* [Doctor, FGD12] to review cases and provide feedback with zero mortalities at their latest review. Many framed the course's value as an investment in broader emergency care capacity that should reach more providers.

## Discussion

This study describes pre-course OERs’ efficacy in an open-access, basic emergency care short course for low-resource settings. The pre-course cases were associated with significantly higher scores on the pre-test, though not at any other time, and did not impact self-efficacy. Nurses experienced greater initial gains and significantly worse retention of knowledge and self-efficacy in comparison to doctors. Despite this, our study demonstrates that BEC has long-term impacts on knowledge and self-efficacy in basic emergency care in accordance with the initial pilot's results in Tanzania.

This study highlights the challenges to OERs and blended learning in LMICs. Our qualitative study found that motivation for self-directed learning, a method requiring learners’ initiative and self-regulation to engage in out-of-classroom studies, significantly correlated with case usage both pre- and post-BEC. Such motivation may determine individual benefit from the cases, or alternatively indicate a higher baseline level of knowledge unrelated to the cases. A recent systematic analysis found that positive interest in online learning, self-efficacy in learning, and constructive workplace environments correlate with self-directed learning readiness and knowledge acquisition among nurses and midwives[24]. Selecting participants with these characteristics and improving workplace learning environments may enhance OERs’ impact in blended learning courses for healthcare professionals.

Our study and others identified cost, time, internet access and technological difficulties as barriers to OER usage in LMICs[7,11,25]. Whereas doctors in SSA tend to have more consistent Internet access and familiarity with OERs[26], nurses in our study reported inconsistent access, technological difficulties and more cost constraints. Though cadre and OER usage had an insignificant relationship in our study, limited or costly internet access may diminish OERs’ benefits among nurses and other providers with less disposable income and technological literacy. Given mobile internet connectivity's expansion to one billion SIM subscribers in Africa by 2025[27], future efforts should focus on incentivising OER-based learning in HCW education as technological barriers lessen. Currently, OERs require substantial implementation measures that may incur more cost than benefit to learners and implementers[12].

Nurses’ significant loss of knowledge and self-efficacy over time compared to doctors’ insignificant decay requires targeted intervention. Nurses reported less clinical training, lower education levels, and fewer opportunities for skill and knowledge application than doctors as they traditionally follow doctors’ orders. Similar studies in East Africa found that doctors and senior medical students retained long-term knowledge and confidence whereas nurses and midwives experienced a decline in both[28,29]. Despite these challenges, nurses demonstrated significant overall improvement in knowledge and self-efficacy retention, gaining the most from the course. As nurses deliver the majority of emergency care in LMICs, we believe that the BEC has the potential to improve emergency care for many patients. Future efforts should focus on nurse-specific interventions to maintain knowledge and skills, including providing nursing mentorship, refresher courses, and BEC-based modules given successful efforts underway in Malawi, Tanzania and Uganda[30].

This study had several limitations. The OER group received the cases before the pre-test, meaning there was no baseline comparison with the control group. The facilitator's presence and inter-cadre hierarchy may have biased FGD dynamics. Convenience sampling may have incurred selection bias. The control and OER groups were not perfectly matched, though timing alone determined this otherwise random assignment. This study did not assess practical skills or behavioural change. The MCQs reflect a representative but limited portion of the BEC's content. We re-administered the same MCQs though participants did not learn the correct answers until the study's conclusion.

Our study provides evidence of the long-term educational impact of the first comprehensive basic and open-access emergency short-course for providers in low-resource settings. Future research should focus on facilitating self-directed learning with OERs, improving nurses’ knowledge retention, and collecting process outcomes in emergency units with BEC-trained staff.

## Dissemination of Results

Preliminary results were presented during the Emergency Medicine Society of South Africa conference in Cape Town, November 2019. The MoH Department of Emergency Medical Services and stakeholders received the study results. The results were further disseminated by authors JK, PK and MO, who are instrumental in BEC coordination and planning in Uganda.

## Authors’ Contribution

Authors contributed as follows to the study's conception or design; the acquisition, analysis, or data interpretation; and drafting or critical revision for important intellectual content: AT, LW, JK and AF contributed 15% each; CC, JB, SS, MO 9% each; and PK 4%. All authors approved the version for publication and agree to be accountable for all aspects of the work.

## Declaration of Competing Interest

Prof Wallis is an editor of the African Journal for Emergency Medicine and Dr. Friedman is a copy editor. Neither participated in this manuscript's editorial process. The journal applies a double blinded process for all manuscript peer review. The authors declared no further conflicts of interest.
